# Our Platform

**Published:** 1912-11

**Authors:** 


### Our Platform

1. The most important matter for the individual physician, the medical journal and the medical society is practical medicine; next medical science.

2. In all questiions of professional ethics and policy, honesty, fairness and efficiency must take precedence of purely theoretic or arbitrary standards.

3. The balance of professional supply and demand must be restored, mainly by inforcing educational requirements.

4. Loyalty to professional organization on the part of the individual physician and vice versa—the latter implying provision for the expression of individual opinions and rule of the majority.

5. Extension of methods of securing professional welfare by cooperation including protection of the individual member by the profession, and care of the disabled and superannuated.

6. Revision of medical nomenclature to avoid the obloquy due to the use of incorrect words—such revision and the supervision of proposed new terms to be relegated to some central and taxation or popular contributions.

7. Control by and in the interests of the profession as a whole, of the medical side of all institutions supported by public taxatioin or popular contributions.

8. Professional organization to accomplish popular instruction and legislation in medical, sanitary and hygienic matters, with due regard to the paramount rights of the community and so as to secure an impartial representation of the profession.

